# Mitochondrial fission as a driver of stemness in tumor cells: mDIVI1 inhibits mitochondrial function, cell migration and cancer stem cell (CSC) signalling

**DOI:** 10.18632/oncotarget.24285

**Published:** 2018-01-19

**Authors:** Maria Peiris-Pagès, Gloria Bonuccelli, Federica Sotgia, Michael P. Lisanti

**Affiliations:** ^1^ Clinical and Experimental Pharmacology Group, Cancer Research UK Manchester Institute, University of Manchester, Manchester, UK; ^2^ Translational Medicine, School of Environment and Life Sciences, Biomedical Research Centre (BRC), University of Salford, Greater Manchester, UK; ^3^ The Paterson Institute, University of Manchester, Withington, UK

**Keywords:** mitochondrial fission, OXPHOS, cancer stem-like cells (CSCs), cell migration, metastasis

## Abstract

Mitochondria are dynamic organelles frequently undergoing fission and fusion events to maintain their integrity, bioenergetics and spatial distribution, which is fundamental to the processes of cell survival. Disruption in mitochondrial dynamics plays a role in cancer. Therefore, proteins involved in regulating mitochondrial dynamics are potential targets for treatment. mDIVI1 is an inhibitor of the mitochondrial fission protein DRP1, which induces i) mitochondrial oxidative stress and ii) effectively reduces mitochondrial metabolism. We show here that mDIVI1 is able to inhibit 3D tumorsphere forming capacity, cell migration and stemness-related signalling in breast cancer cells, indicating that mDIVI1 can potentially be used for the therapeutic elimination of cancer stem cells (CSCs).

## INTRODUCTION

Mitochondria are extremely dynamic organelles in constant division, elongation and connection to each other to form tubular networks or fragmented granules in order to satisfy the requirements of the cell and adapt to the cellular microenvironment. The balance of mitochondrial fusion and fission dictates the morphology, abundance, function and spatial distribution of mitochondria, therefore influencing a plethora of mitochondrial-dependent vital biological processes such as ATP production, mitophagy, apoptosis and calcium homeostasis [1]. In turn, mitochondrial dynamics can be regulated by mitochondrial metabolism, respiration and oxidative stress [2]. Thus, it is not surprising that an imbalance of fission and fusion activities has a negative impact on several pathological conditions, including cancer. Cancer cells often exhibit fragmented mitochondria, and enhanced fission or reduced fusion is often associated with cancer, although a comprehensive mechanistic understanding on how mitochondrial dynamics affects tumorigenesis is still needed [3].

A master regulator of the mitochondrial fission machinery is the cytoplasmic dynamin-related protein 1 (DRP1, also known as DNM1L). During mitochondrial fission DRP1 is recruited to the mitochondria, where it interacts with its outer mitochondrial membrane receptors. Subsequently, DRP1 polimerizes and promotes mitochondrial constriction and fission, activities that are fuelled by its GTPase activity [4].

**Figure 1:**
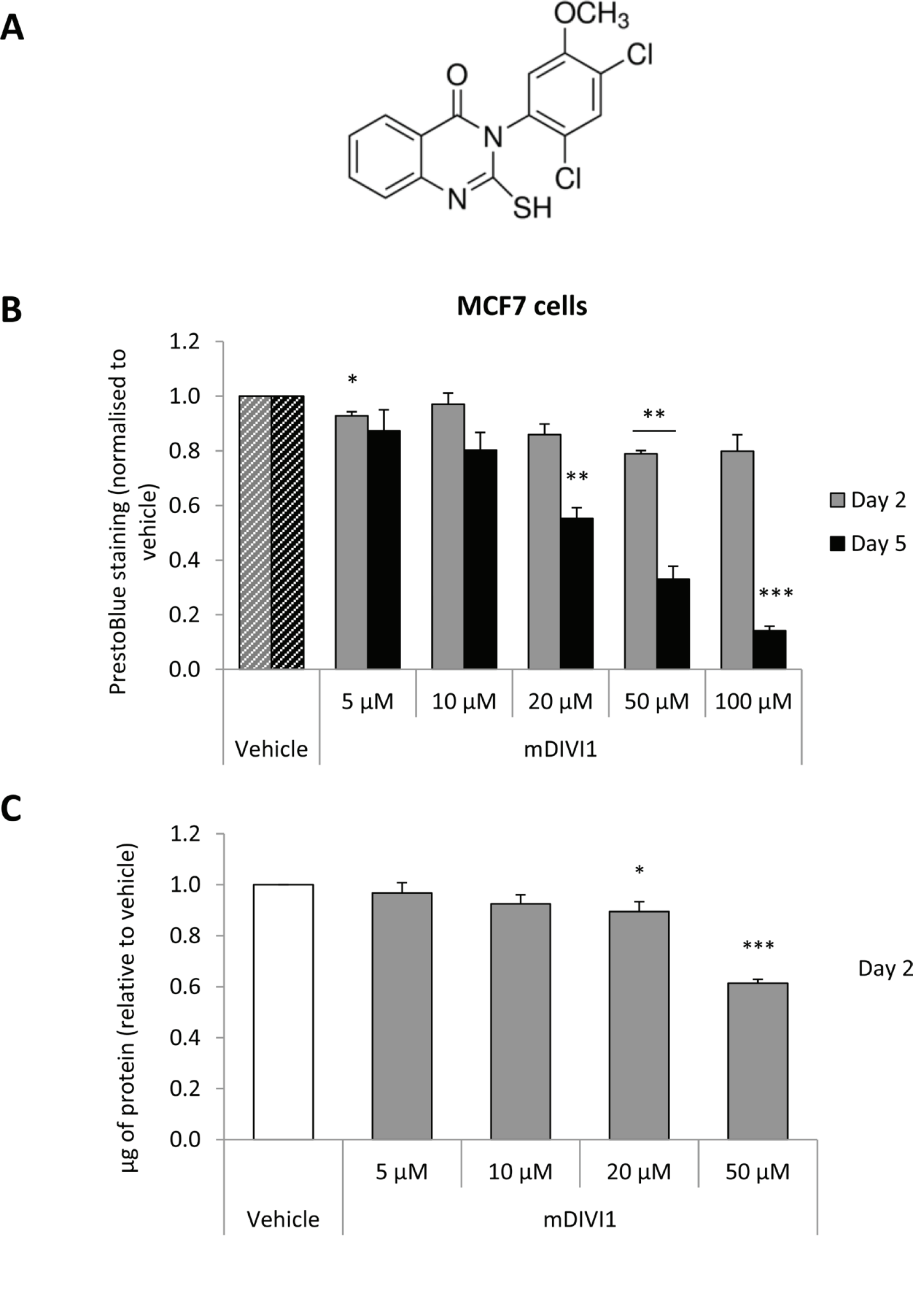
DRP1 inhibition by mDIVI1 reduces MCF7 cell viability (**A**) mDIVI1 chemical structure (**B**) The mitochondrial fission inhibitor mDIVI1 did not decrease viability of MCF7 cells at a concentration of 10 µM, and significantly reduced it at 50 µM and 100 µM at 48 hours. Five days of treatment induced a bigger reduction of MCF7 viability (*N* = 3). (**C**) mDIVI1 slightly decreased protein content of MCF7 cells at a concentration of 10 µM and reduced it by almost 40% at 50 µM after 48 hours of treatment as measured using the Bradford assay (*N* = 6). Data represented as MEAN ± SEM. One sample *t* test, ^*^p value < 0.05, ^**^p value < 0.01 and ^***^p value < 0.001.

The discovery of a derivative of quinazolinone, the mitochondrial DIVision Inhibitor 1 (mDIVI1), a small molecule that selectively and reversibly inhibits DRP1 [5] (Figure 1A), has helped to shed new light on the role of mitochondrial dynamics in cancer. mDIVI1 has been shown to target DRP1 by binding and suppressing both the DRP1 self-assembly into ring-like structures around the mitochondria and its capacity to catalyze GTP hydrolysis. mDIVI1 prompts a rapid formation of interconnected mitochondria without overtly affecting other cellular structures such as the cytoskeleton or the endoplasmic reticulum. The IC50 of mDIVI1 ranges from 1 to 50 µM depending on the cell type [5]. In contrast to the cytoprotective effect in neurons and cardiovascular cells, mDIVI1 has a cytotoxic effect in hyperproliferative cancer cells and immortalised cell lines [4]. Indeed, high DRP1 expression or activation has been described in several malignancies, and it promotes mitochondrial fission in cancer cells, which plays an important role in their proliferation and metastatic capacity [4]. Reversal of that mitochondrial fission via DRP1 inhibition with mDIVI1 induces apoptosis via cytochrome c release and cell cycle arrest by impairing the assembly of mitotic spindles and cytokinesis, consequently leading to aneuploidy [3, 4].

Finally, emerging evidence points toward a role for mitochondrial fusion and fission, and in particular for DRP1, in regulating the proliferation and survival of cancer stem cells (CSC), which are thought to be responsible for treatment failure and metastatic dissemination. DRP1-dependent fission confers chemoresistance, as chemoresistant cancer cells are prone to form highly interconnected mitochondrial networks. mDIVI1 treatment reverses this phenotype by re-sensitising chemoresistant cancer cells [6]. Moreover, high DRP1 expression and mitochondrial fragmentation contribute to maintenance of brain tumour-initiating cells, and genetic ablation of DRP1 or its pharmacological inhibition with mDIVI1 reduces their tumorigenicity *in vitro* and *in vivo* [7]. Of note, DRP1-dependent fission has been found to be essential for stem cell maintenance in immortalised mammary epithelial stem-like cells. Upon asymmetric cell division, stem-like cells contained a higher abundance of newly generated mitochondria, whereas cells with more aged mitochondria were growing less efficiently in anchorage-independent conditions and were primed to differentiate. DRP1 inhibition with mDIVI1 abolished the mitochondrial asymmetric distribution of mitochondria reducing stem-cell properties *in vitro*, suggesting that mitochondrial fitness regulates stemness [8]. Finally, inactivation of DRP1 with mDIVI1 seems to impede pluripotency reprogramming [9, 10]. Specific eradication of CSCs represents one of the most challenging goals of current cancer research, as it could potentially achieve a permanent cure for cancer patients.

Emerging evidence has recently shown that CSCs are critically dependant on mitochondrial function for their successful propagation and survival [11–13]. In the current study, we hypothesised that the pharmacological inhibition of DRP1-induced mitochondrial fission with mDIVI1 would target breast CSC survival via induction of mitochondrial dysfunction and repression of mitochondrial metabolism, extending our previous studies. We show that exposure of MCF7 breast cancer cells to mDIVI1 transforms these cells in metabolically less active cells, with minor ATP demand, possibly via induction of mitochondrial reactive species. These mDIVI1-treated metabolically-repressed MCF7 cells show reduced tumorsphere forming capacity, decreased migration and inhibited stemness-related signalling. mDIVI1 is also able to decrease tumorsphere formation efficiency in melanoma and lung cancer cell lines, strongly suggesting that mDIVI1 treatment decreases the abundance of CSC also in these cancers, hence pointing it out as a new anti-CSC drug for cancer therapy.

## RESULTS

In this study we aimed to assess the effects of mDIVI1, an inhibitor of the mitochondrial division protein DRP1, on mitochondrial function and CSC behaviour.

### MDIVI1 treatment reduces MCF7 cell viability

First of all, we sought to evaluate whether mDIVI1-induced inhibition of DRP1 would have a repercussion on the viability of MCF7 cells. Exposure to mDIVI1 for 48 hours did not decrease viability of MCF7 cells at a concentration of 10 µM, and significantly reduced it by 20% at 50 µM and 100 µM, as measured using the PrestoBlue assay, a resazurin-based viability assay (Figure 1B). Similar results were obtained using the Bradford assay, which measures protein content, as an indicator of cell viability (Figure 1C). Only exposure to higher concentrations of mDIVI1 (20 and 50 µM) significantly reduced MCF7 protein content after 2 days of treatment. Five days of treatment did have a bigger impact on MCF7 cell viability, significantly diminishing it by 20% at a concentration of 10 µM, by over 65% at 50 µM and 85% at 100 µM (Figure 1B).

Thus, mDIVI1 reduces the viability of MCF7 cells mostly at higher concentrations and after 5 days of treatment.

### MDIVI1 increases MCF7 mitochondrial mass and mitochondrial oxidative stress

**Figure 2:**
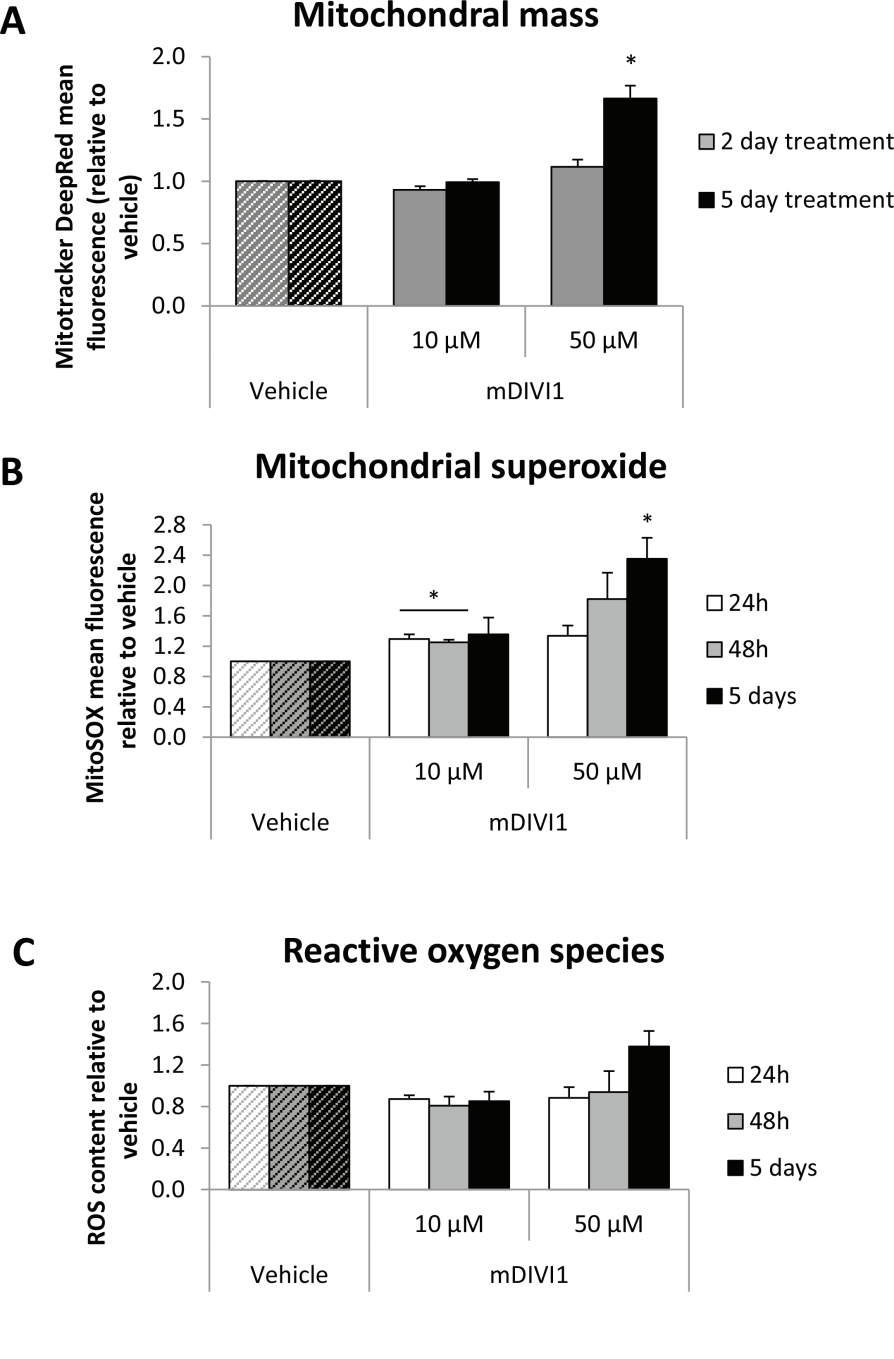
Treatment of MCF7 cells with mDIVI1 significantly increases their mitochondrial mass and mitochondrial oxidative stress at 50 µM (**A**) Mitotracker Deep Red mean intensity significantly increased after 5 days of treatment with 50 µM of mDIVI1. (**B**) Mitochondrial superoxide production significantly increased after 24 hour, 2 and 5 days of treatment. (**C**) Total ROS levels were not altered after exposure to mDIVI1. Data represented as MEAN ± SEM. One sample *t* test, ^*^p value < 0.05, ^**^p value < 0.01 and ^***^p value < 0.001. *N* = 3.

In order to assess whether the inhibition of DRP1 was actually being translated into a reduction in mitochondrial fission, we aimed to stain the mitochondria of MCF7 cells with Mitotracker Deep Red, which is a marker of mitochondrial mass. mDIVI1 treatment led to more interconnected mitochondria, with slightly increased mitochondrial calibres, which could be a result of the absence of fission activity (Supplementary Figure 1). Mitochondrial mass was also quantified by flow cytometry. Mitotracker Deep Red mean fluorescence intensity was found to be significantly higher after 5 days of treatment with 50 µM mDIVI1, indicating that at higher concentrations, mDIVI1-mediated DRP1 inhibition is able to increase the mitochondrial mass of MCF7 cells (Figure 2A).

We hypothesised that an inhibition of the mitochondrial fission would have an impact on other mitochondrial processes such as mitochondrial metabolism and general and mitochondrial oxidative stress. To test that, MCF7 cells were stained with MitoSOX and CM-H2DCFDA, and mitochondrial superoxide and total ROS were quantified by flow cytometry. MitoSOX staining quantification in MCF7 cells revealed that exposure to both concentrations of mDIVI1 significantly increased mitochondrial superoxide production compared to vehicle-treated cells (Figure 2B). However, general oxidative stress levels did not change after exposure to mDIVI1. Only 5 days of treatment showed a slight trend toward an increase in the production of total ROS (Figure 2C). Of note, whereas the increase in general ROS goes in line with the increase in mitochondrial content, the boost in the levels of mitochondrial superoxide in mDIV1-treated cells is actually bigger than the observed increased mitochondrial content.

Thus, mDIVI1 treatment slightly increase mitochondrial mass and clearly induced the generation of mitochondrial superoxide without any major effects on MCF7 general oxidative stress.

### MDIVI1 reduces glycolytic capacity, respiration and ATP production of MCF7 cells

**Figure 3:**
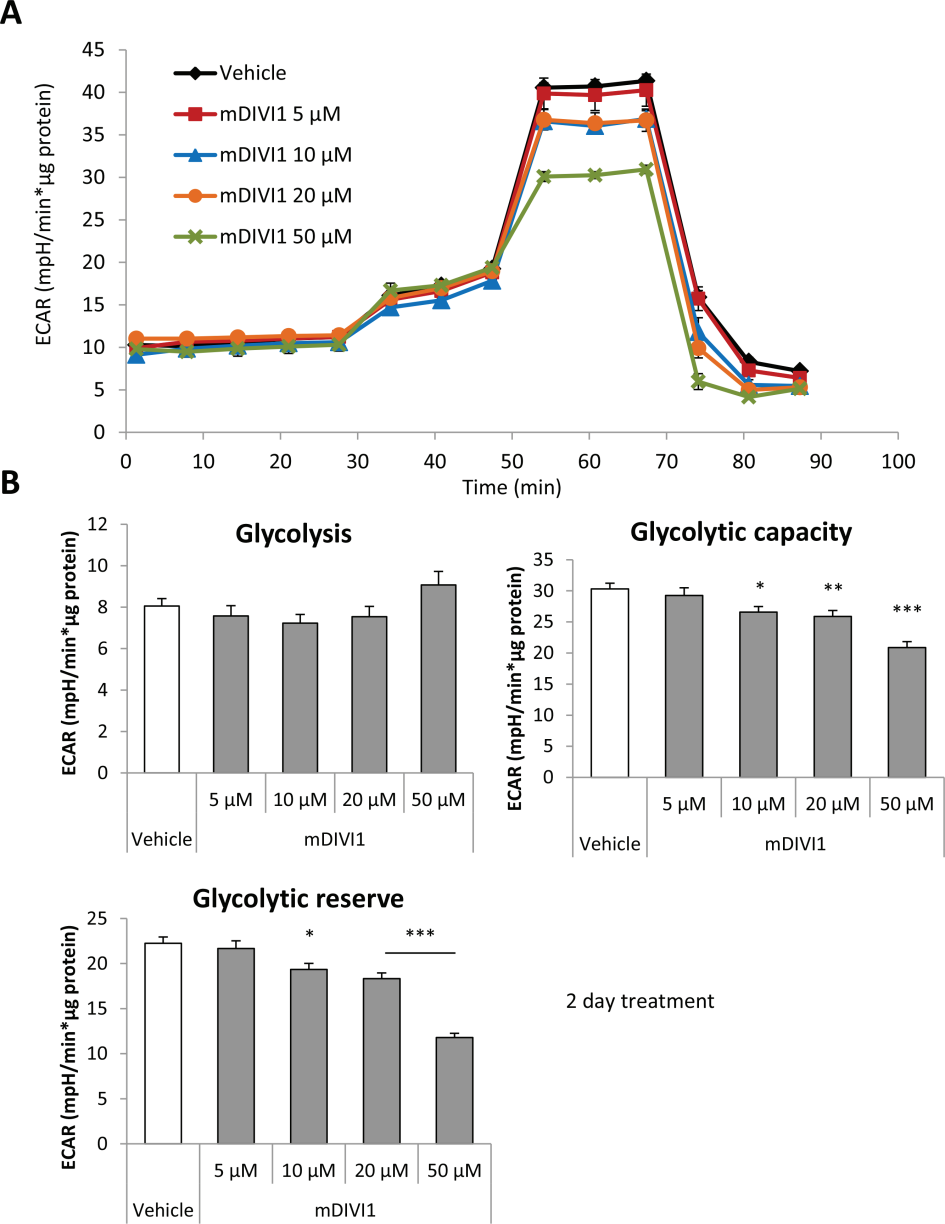
mDIVI1 treatment significantly reduces the glycolytic capacity of MCF7 cells without having an effect on basal glycolysis, hence decreasing their glycolytic reserve (**A**) Extracellular acidification rate (ECAR) of MCF7 cells treated with either vehicle or mDIVI1 for 48 hours and assessed using the Seahorse XFe96 Analyzer. (**B**) Treatment with ≥10 µM mDIVI1 for 48 hours blocks the increase of ECAR usually linked to oligomycin inhibition of the mitochondrial respiration, indicating a significant reduction in glycolytic capacity and reserve. mDIVI1-treated cells are using glycolysis closer to its maximum capacity compared to vehicle-treated cells. Data represented as MEAN ± SEM. One way ANOVA, Dunnett’s multiple comparisons test, ^*^p value < 0.05, ^**^p value < 0.01 and ^***^p value < 0.001. *N* ≥ 3.

**Figure 4:**
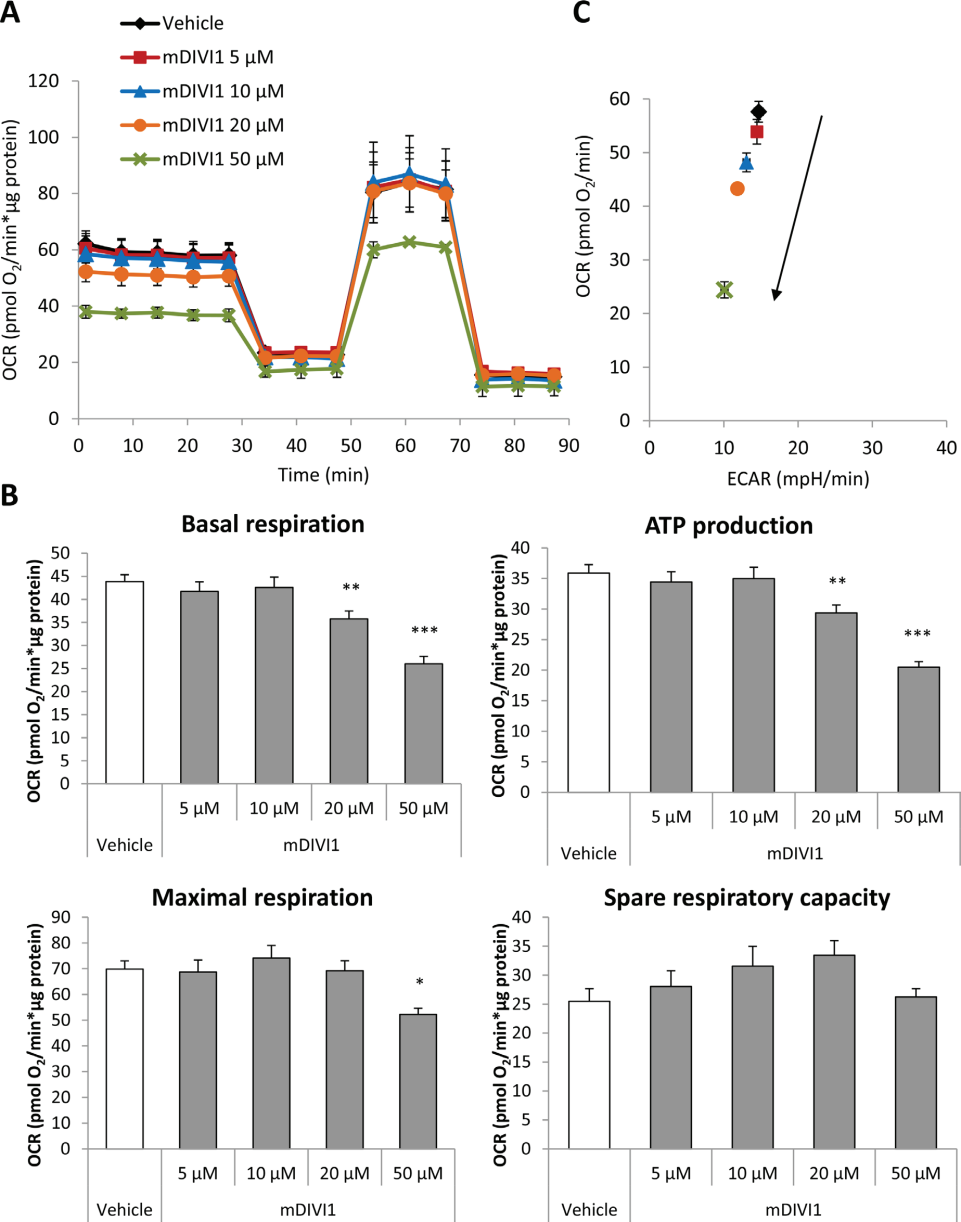
mDIVI1 treatment significantly reduces the respiration and ATP production of MCF7 cells but increases their spare respiratory capacity (**A**) Oxygen consumption rate (OCR) of MCF7 cells treated with either vehicle or mDIVI1 for 48 hours and assessed using the Seahorse XFe96 Analyzer. (**B**) Treatment with mDIVI1 for 48 hours decreases the oxygen consumption linked to basal respiration, ATP production and to a lesser extent, maximal respiration. However it increases the spare respiratory capacity of MCF7cells, suggesting that mDIVI1-treated cells may be able to manage better an energetic crisis. (**C**) mDIVI1 treatment decreases the OCR/ECAR ratio of MCF7 cells indicating that mDIVI1-treated MCF7 cells are less aerobic and less metabolically active. Data represented as MEAN ± SEM. One way ANOVA, Dunnett’s multiple comparisons test, ^*^p value < 0.05, ^**^p value < 0.01 and ^***^p value < 0.001. *N* = 3.

We hypothesised that inhibition of mitochondrial fission would be enough to block the normal functioning of mitochondrial metabolism. Indeed, it has been shown that a DRP1 mutant that inhibits mitochondrial fission increases glucose uptake and lactate production, and decreases ATP production [14]. Thus, we next aimed to measure the glycolytical function and the mitochondrial respiration in MCF7 cells exposed to mDIVI1. The extracellular acidification rate (ECAR) and the oxygen consumption rate (OCR) were measured using an XF96 Extracellular Flux Analyser (Figures 3A and 4A). Basal glycolysis, glycolytic capacity and glycolytic reserve were calculated after addition of glucose, oligomycin and 2-deoxyglucose (2DG) into the media. Surprisingly, exposure to mDIVI1 did not have a significant effect on basal glycolysis. However, the glycolytic capacity and glycolytic reserve of MCF7 cells was reduced after treatment with mDIVI1 (Figure 3B). That is, treatment with mDIVI1 for 48 hours blocked the increase of the ECAR usually linked to the oligomycin-induced inhibition of mitochondrial complex V of the electron transport chain, indicating that mDIVI1-treated MCF7 either have less ATP demand or have a less efficient mitochondrial oxidative phosphorylation than vehicle-treated cells. Thus, to measure basal respiration, ATP production, maximal respiration and spare respiratory capacity, oxygen consumption was also calculated after addition of oligomycin, FCCP and antimycin/rotenone into glucose-containing media. In fact, exposure to mDIVI1 for 48 hours significantly reduced the oxygen consumption linked to basal respiration, ATP production and to a lesser extent, maximal respiration at higher concentrations (Figure 4B). However, it slightly increased the spare respiratory capacity of MCF7 cells after treatment with all mDIVI1 concentrations, suggesting that basal respiration in mDIVI1-treated is further from its theoretical maximum than vehicle-treated cells. The OCR versus ECAR graph was also plotted to have an indication of the metabolic state of the cell. mDIVI1 treatment also decreased dose-dependently the OCR/ECAR ratio of MCF7 cells compared to vehicle, indicating that mDIVI1-treated MCF7 cells are less aerobic and metabolically less active (Figure 4C). Thus, mDIVI1-induced inhibition of mitochondrial fission functionally targets oxidative phosphorylation and also the glycolytic function of MCF7 cells, transforming them into cells with lower mitochondrial energetic needs.

To identify differentially regulated proteins upon treatment with mDIVI1, MCF7 cells were exposed for 48 hours to either vehicle or 10 µM mDIVI1 and cell lysates were subject to label-free quantitative proteomics. Following protein digestion with trypsin, peptide fractions were processed on an LTQ-Orbitrap XL mass spectrometer. Those peptides identified were further analysed to find proteomic changes between mDIVI1-treated and vehicle-treated MCF7 cells, as described before [15]. To define differential regulation, those identified proteins that showed a fold change difference of 1.5 or higher, and *p* values of < 0.05 (ANOVA) compared to vehicle were considered. First, we searched the proteomics datasets for changes in proteins involved in metabolism (summarised in Table 1). The expression of several glycolytic enzymes, as well as pentose phosphate pathway enzymes and enzymes involved in mitochondrial metabolism were found to be down-regulated in mDIVI1-treated MCF7 cells compared to vehicle treatment. Of note, hexokinase, which generates glucose 6-phosphate from glucose for the glycolytic and the pentose phosphate pathways, was infinitely down-regulated. In fact, the first two enzymes of the oxidative branch of the pentose phosphate pathway were also found downregulated in mDIVI1-treated cells compared to vehicle-treated cells (Table 1). That may possibly translate into a loss of antioxidant power, as the pentose phosphate pathway is a major source of NADPH. Furthermore, components of the complex III and V of the electron transport chain were found to be highly downregulated in mDIVI1-treated MCF7 cells (Table 1). To obtain additional functional insights into pathways that are differentially regulated in mDIVI1-treated MCF7 cells, bioinformatics analysis of the proteomics datasets were conducted. All proteins were analysed using the Ingenuity Pathway Analysis software (IPA) to seek altered canonical pathways and toxicity functions. Of note, amongst the altered pathways identified by IPA in the mDIVI1-treated cells were pathways involved in metabolism such as pentose phosphate pathway, mTOR signalling, AMPK and protein kinase A signalling, fatty acid biosynthesis, cholesterol and palmitate biosynthesis, mevalonate pathway and ketolysis. Likewise, the toxicity functions found to be altered in mDIVI1-treated MCF7 cells compared with vehicle treatment included decreases in permeability transition of mitochondria and mitochondrial membrane, fatty acid metabolism and cholesterol biosynthesis (Table 2). Thus, besides mitochondrial respiration and glycolysis, fatty acid metabolism also seems to be altered in mDIVI1-treated MCF7 cells, as observed by label-free quantitative proteomics.

**Table 1:** Changes in the expression of enzymes involved in several cellular metabolic pathways after exposure of MCF7 cells to mDIVI1 for 48 hours as measured by label-free quantitative proteomics

Glycolysis		10 µM mDIVI1
Hexokinase 1	HK1	↓Infinite
Fructose-bisphosphate aldolase A	ALDOA	↓ 2.88
Enolase 1	ENO1	↓ 6.63
**Post-Glycolysis Processes**		
Pyruvate dehydrogenase	PDHB	↑ 1.63
**Pentose Phosphate Pathway**		
6-phosphogluconolactonase	PGLS	↓ 1.56
Phosphogluconate dehydrogenase	PGD	↓ 3.91
Transketolase		
**TCA Cycle**	TKT	↑ 2.06
Citrate synthase, mitochondrial	CS	↓ 1.51
Isocitrate dehydrogenase	IDH2	↑ 1.84
	IDH3G	↓ 1.51
**Oxidative Phosphorylation**		
NADH dehydrogenase (complex I) MT-ND5	NDUFV1	↓ 1.63
	↑ 1.84	
Coenzyme Q – cytochrome c reductase (complex III)	UQCRC1	↓ 2569.35
Cytochrome c oxidase (complex IV)	COX6A1	↑ 1.70
ATP synthase (complex V)	ATP5O	↓ 13.56
**Fatty Acid Oxidation**		
Acetyl-Coenzyme A acyltransferase 1, peroxisomal	ACAA1	↑ 3.96
Acetyl-Coenzyme A acyltransferase 2, mitocondrial	ACAA2	↓ 2.09
Long-chain-aldehyde dehydrogenase	ALDH3A2	↑ 4.40
Delta-1-pyrroline-5-carboxylate dehydrogenase, mitochondrial	ALDH4A1	↑ 2.40
4-trimethylaminobutyraldehyde dehydrogenase	ALDH9A1	↑ 1.72
Acyl-CoA dehydrogenase family member 9, mitochondrial	ACAD9	↓ 1.94
Carnitine O-palmitoyltransferase 2, mitochondrial	CPT2	↑ 3.15
**Fatty Acid Synthesis**		
Fatty acid synthase	FASN	↑ 1.97
Beta-ketoacyl-ACP synthase	OXSM	↓ 1.61
**Ketolysis/Ketogenesis**		
Acetyl-CoA acetyltransferase, mitochondrial	ACAT1	↓ 2.40
3-oxoacid CoA-transferase 1, mitochondrial	OXCT1	↑ 1.64
3-hydroxy-3-methylglutaryl-CoA synthase 1	HMGCS1	↑ 4.09
**Lipid Metabolism (Other)**		
short/branched chain specific acyl-CoA dehydrogenase	ACADSB	↓ 2.92
Cytochrome P450 1B1	CYP1B1	↑ 19.96
Cytochrome P450, family 1, subfamily A, polypeptide 1	CYP1A1	↑ 190.30
Lanosterol 14 α-demethylase	CYP51A1	↑ 3.41
Lanosterol synthase	LSS	↓ 1.82
Farnesyl-diphosphate farnesyltransferase 1	FDFT1	↓ 1.50
Isopentenyl-diphosphate delta isomerase	IDI1	↓ 1.79

**Table 2:** Pathway analysis of differentially expressed proteins in MCF7 cells treated with mDIVI1 compared to vehicle-treated cells

Ingenuity canonical pathways	-log (*p*-value)	Z score	Ratio
Remodeling of Epithelial Adherens Junctions	13.6	−0.30	0.348
Actin Cytoskeleton Signaling	11.5	−1.15	0.176
Germ Cell-Sertoli Cell Junction Signaling	9.79		0.183
EIF2 Signaling	9.4	−0.83	0.173
Epithelial Adherens Junction Signaling	8.9		0.189
Tight Junction Signaling	8.68		0.175
Integrin Signaling	8.46	−0.36	0.156
ILK Signaling	8.38	0.73	0.161
Regulation of eIF4 and p70S6K Signaling	8.17	−1.41	0.175
Paxillin Signaling	7.85	−0.69	0.2
Caveolar-mediated Endocytosis Signaling	7.42		0.239
Sertoli Cell-Sertoli Cell Junction Signaling	7.07		0.156
Regulation of Actin-based Motility by Rho	6.77	−0.73	0.207
Phagosome maturation	6.61		0.178
Clathrin-mediated Endocytosis Signaling	6.48		0.143
Regulation of Cellular Mechanics by Calpain Protease	5.75	−2.24	0.236
RhoA Signaling	5.26	−0.94	0.158
Mitotic Roles of Polo-Like Kinase	5.05	0.82	0.206
RhoGDI Signaling	4.95	0.85	0.134
Protein Ubiquitination Pathway	4.71		0.114
Role of BRCA1 in DNA Damage Response	4.66		0.179
Leukocyte Extravasation Signaling	4.62	−0.63	0.122
mTOR Signaling	4.53	0.63	0.123
Actin Nucleation by ARP-WASP Complex	4.15	−1.51	0.196
FAK Signaling	4.1		0.153
DNA Double-Strand Break Repair by Non-Homologous End Joining	3.42		0.357
Signaling by Rho Family GTPases	3.35	−0.41	0.102
Estrogen Receptor Signaling	3.29		0.125
Superpathway of Cholesterol Biosynthesis	2.79		0.222
Cleavage and Polyadenylation of Pre-mRNA	2.69		0.333
AMPK Signaling	2.66	−0.63	0.101
Palmitate Biosynthesis I	2.62		1
Fatty Acid Biosynthesis Initiation II	2.62		1
Calcium Signaling	2.39	−0.81	0.1
Cdc42 Signaling	2.37	−0.83	0.109
Rac Signaling	2.36	−0.83	0.112
Telomere Extension by Telomerase	2.3		0.267
RAN Signaling	2.19		0.25
Assembly of RNA Polymerase I Complex	2.11		0.333
ERK/MAPK Signaling	2.07	−0.47	0.0909
Cell Cycle Control of Chromosomal Replication	2.04		0.185
PAK Signaling	2.02	−0.30	0.11
Protein Kinase A Signaling	2.01	−1.61	0.0775
Apoptosis Signaling	1.97	−1.26	0.114
Pentose Phosphate Pathway	1.97		0.3
Death Receptor Signaling	1.88	−1.26	0.11
Unfolded protein response	1.85		0.132
DNA Methylation and Transcriptional Repression Signaling	1.83		0.2
Mevalonate Pathway I	1.74		0.25
Ephrin Receptor Signaling	1.67	−1.39	0.0872
Agranulocyte Adhesion and Diapedesis	1.61		0.0857
GM-CSF Signaling	1.59	−0.38	0.11
Axonal Guidance Signaling	1.56		0.0703
Gap Junction Signaling	1.52		0.0854
Ketolysis	1.37		0.286
CDK5 Signaling	1.3	−1.34	0.0918

### MDIVI1 reduces tumorsphere formation in MCF7 breast cancer, A375 melanoma and A549 lung cancer cell lines

As mDIVI1 functioned as an inhibitor of mitochondrial oxidative phosphorylation in MCF7 breast cancer cells, we next examined the effects of this mitochondrial fission inhibitor on the behaviour of CSCs, as it has been previously described that CSCs depend on mitochondrial metabolism for their survival and propagation [11–13].

**Figure 5:**
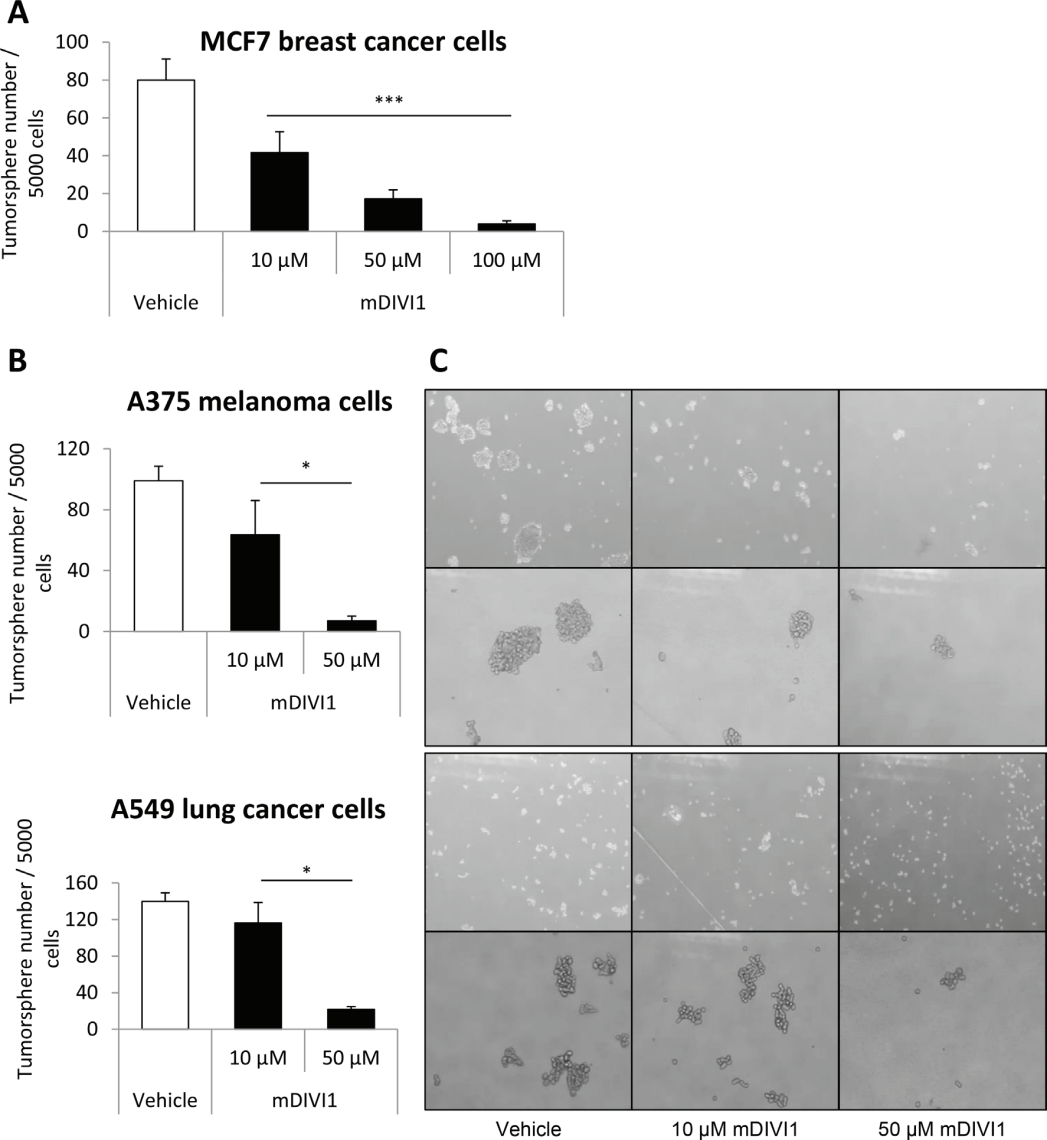
mDIVI1 treatment significantly reduces MCF7 tumorsphere formation, as well as the formation of A375 melanoma and A549 lung tumorspheres (**A**) Five days of treatment with the mitochondrial fission inhibitor mDIVI1 decreased tumorsphere number by 50% at a concentration of 10 µM by 80% at 50 µM and 95% at 100 µM. One-way ANOVA, post test for linear trend, ^***^p value < 0.001. *N* = 3. (**B**) Five days of treatment with the mitochondrial fission inhibitor mDIVI1 dose-dependently decreased tumorsphere formation efficiency also in a melanoma (A375) and a lung cancer (A549) cell line. Data represented as MEAN ± SEM. One-way ANOVA, post test for linear trend ^*^p value < 0.05 *N* = 2. (**C**) Representative image of A375 melanoma and A549 lung tumorspheres after treatment with either vehicle or mDIVI1. Inhibition of DRP1 with mDIVI1 decreased tumorsphere number and also tumorsphere size.

One of the gold standard techniques for the study and identification of CSCs is the sphere formation assay, an *in vitro* culture system that enriches for CSCs. Under these anchorage-independent culture conditions, CSCs preferentially form the so-called tumorspheres, whereas more differentiated cells die rapidly. Hence, MCF7 cells were grown in suspension as tumorspheres and treated with either vehicle or mDIVI1 at increasing concentrations. Five days of exposure to mDIVI1 decreased MCF7 breast tumorsphere number by 50% at a concentration of 10 µM by 80% at 50 µM and 95% at 100 µM compared to vehicle treated cells (Figure 5A). Likewise, A375 melanoma and A549 lung tumorsphere forming capacity were significantly diminished after treatment with mDIVI1, again in a dose-dependent fashion (by over 35% and 15% at a concentration of 10 µM and by over 90% and 80% at 50 µM, respectively) (Figure 5B and 5C). Inhibition of DRP1 with mDIVI1 seemed to not only decrease tumorsphere number but also tumorsphere size (Figure 5C). Thus, mDIVI1 inhibited tumorsphere formation in a dose-dependent manner not only in the breast cancer cell line MCF7, but also in melanoma (A375) and lung cancer (A549) cell lines.

### MDIVI1 inhibits migration of MCF7 cells

**Figure 6:**
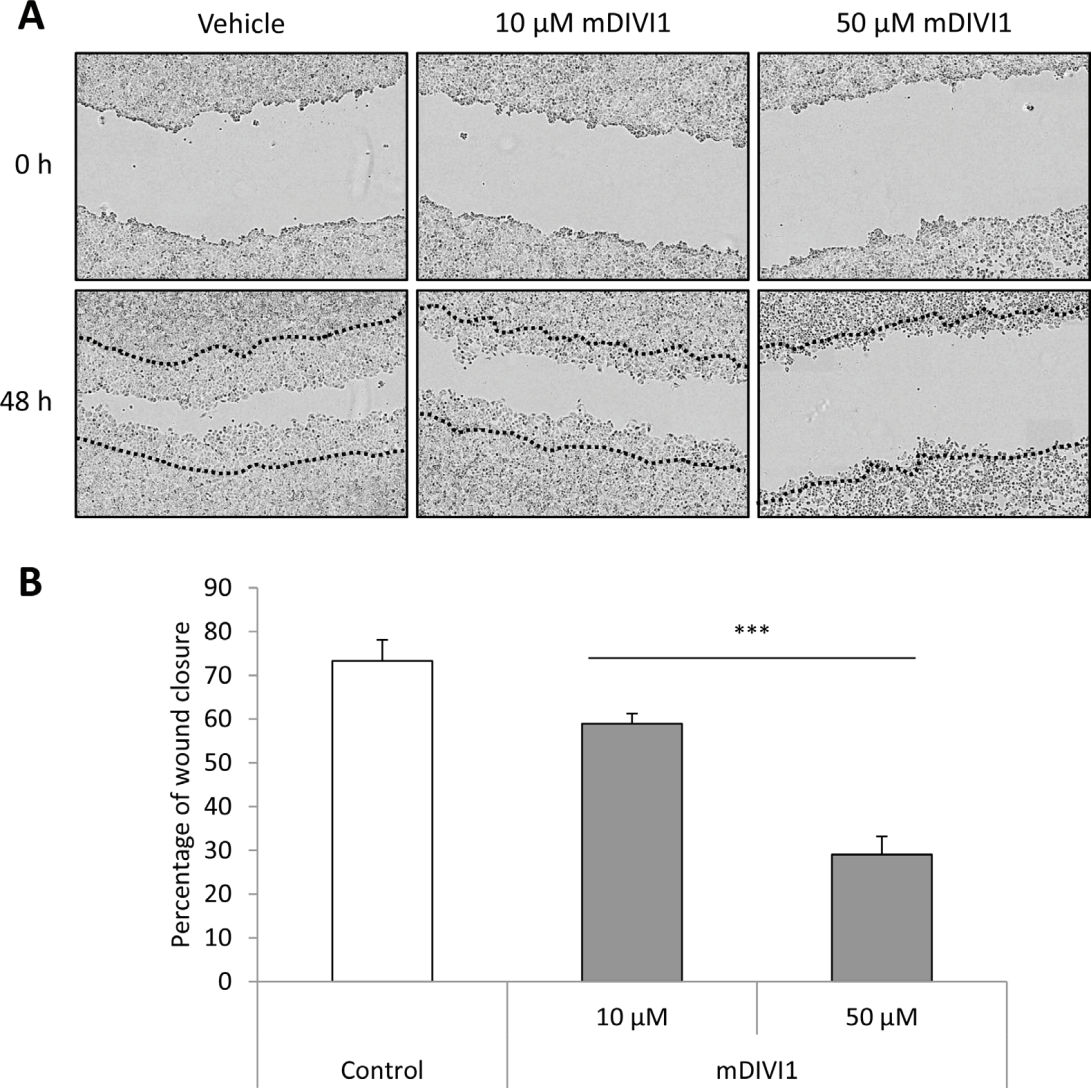
mDIVI1 treatment significantly reduces MCF7 migration *in vitro* (**A**) Representative image of MCF7 cells after treatment with either vehicle or mDIVI1. Inhibition of DRP1 with mDIVI1 reduces scratch closure *in vitro*. (**B**) Two days of treatment with the mitochondrial fission inhibitor mDIVI1 significantly decreased the percentage of wound closure. Data represented as MEAN ± SEM. One-way ANOVA, post test for linear trend ^***^p value < 0.001. *N* = 4.

It is though that CSCs are responsible for metastatic spread and growth. Emerging evidence highlights the role of mitochondrial dynamics in tumour cell dissemination [3]. These studies suggest that mitochondria are transferred to sites with high-energy demand. In migrating cancer cells mitochondria accumulate at the leading edge where processes requiring high energy occur, such as formation of focal adhesions. Fission seems to be a prerequisite for that efficient relocation of mitochondria. Indeed, upregulation/activation of DRP1 is associated with a migratory and invasive phenotype in cancer [3]. Thus, we next sought to assess whether mDIVI1-induced inhibition of DRP1 would decrease the migration of MCF7. To that purpose we performed a scratch assay, were cells were cultured in the presence of either vehicle or mDIVI1. The scratch was monitored over time and the percentage of wound closure was measured as described previously [16]. Exposure to 10 and 50 µM mDIVI1 significantly reduced the percentage of wound closure compared to vehicle-treated cells on a 14% and 44% respectively, therefore indicating a clear mDIVI1-induced dose-dependent reduction in MCF7 cell migration (Figure 6).

In addition, amongst the altered pathways identified by IPA in the mDIVI1-treated cells, there were pathways involved in cell motility such as actin cytoskeleton signalling, regulation of actin-based motility by rho, regulation of cellular mechanics by calpain protease (clearly predicted to be inactivated), cdc42 or RhoA signalling, and PAK signalling (Table 2). Likewise, some of the biological functions identified by IPA as affected by mDIVI1 involved cellular movement, migration and invasion, which in most cases was predicted to be decreased (Table 3).

**Table 3:** Toxicity effects of differentially expressed proteins and biological functions affected by mDIVI1 treatment compared to vehicle-treated MCF7 cells

Categories	Biological functions	*p*-Value	Activation z-score
Cellular movement	Invasion of cells	0.00000127	−1.806
	Invasion of tumour cell lines	0.00000205	−1.652
	Cell movement of tumour cell lines	0.0000138	−1.628
	Cell movement	0.0000841	−1.295
	Migration of tumour cell lines	0.000153	−1.164
	Cell movement of breast cancer cell lines	0.000655	−1.968
Cell Cycle, Cellular Movement	Cytokinesis	0.0000132	−0.669
Cell Cycle, DNA Replication, Recombination, and Repair	Recombination of cells	0.0000153	−1.732
	Homologous recombination of cells	0.0000327	−1.732
	Homologous recombination	0.000264	−1.387
Cell Cycle	Cell cycle progression	0.000218	−1.762
Cell Morphology, Cellular Function and Maintenance, DNA Replication, Recombination, and Repair	Repair of cells	0.00145	1.921
	Double-stranded DNA break repair of cells	0.0035	2.069
Cell Morphology	Shape change of tumour cell lines	0.00354	−1.446
	Cell spreading	0.00383	−1.633
Cellular Assembly and Organization	Formation of cytoskeleton	0.000722	1.95
	Formation of nucleus	0.00185	−2
Cellular Function and Maintenance	Endocytosis	0.000264	−1.832
Cellular Assembly and Organization	Formation of cytoskeleton	0.000722	1.95
Gene Expression	Transcription	0.000829	1.993
DNA Replication, Recombination, and Repair	Double-stranded DNA break repair	0.000932	2.123
Cell Death and Survival	Cell viability	0.00417	−2.813
**Ingenuity Toxicity Lists**		**-log (*p*-value)**	**Ratio**
Cholesterol Biosynthesis		3.12	0.312
Fatty Acid Metabolism		3.02	0.133
Decreases Permeability Transition of Mitochondria and Mitochondrial Membrane		2.46	0.429

### MDIVI1 inhibits signalling pathways required for CSC survival in MCF7 cells

**Figure 7:**
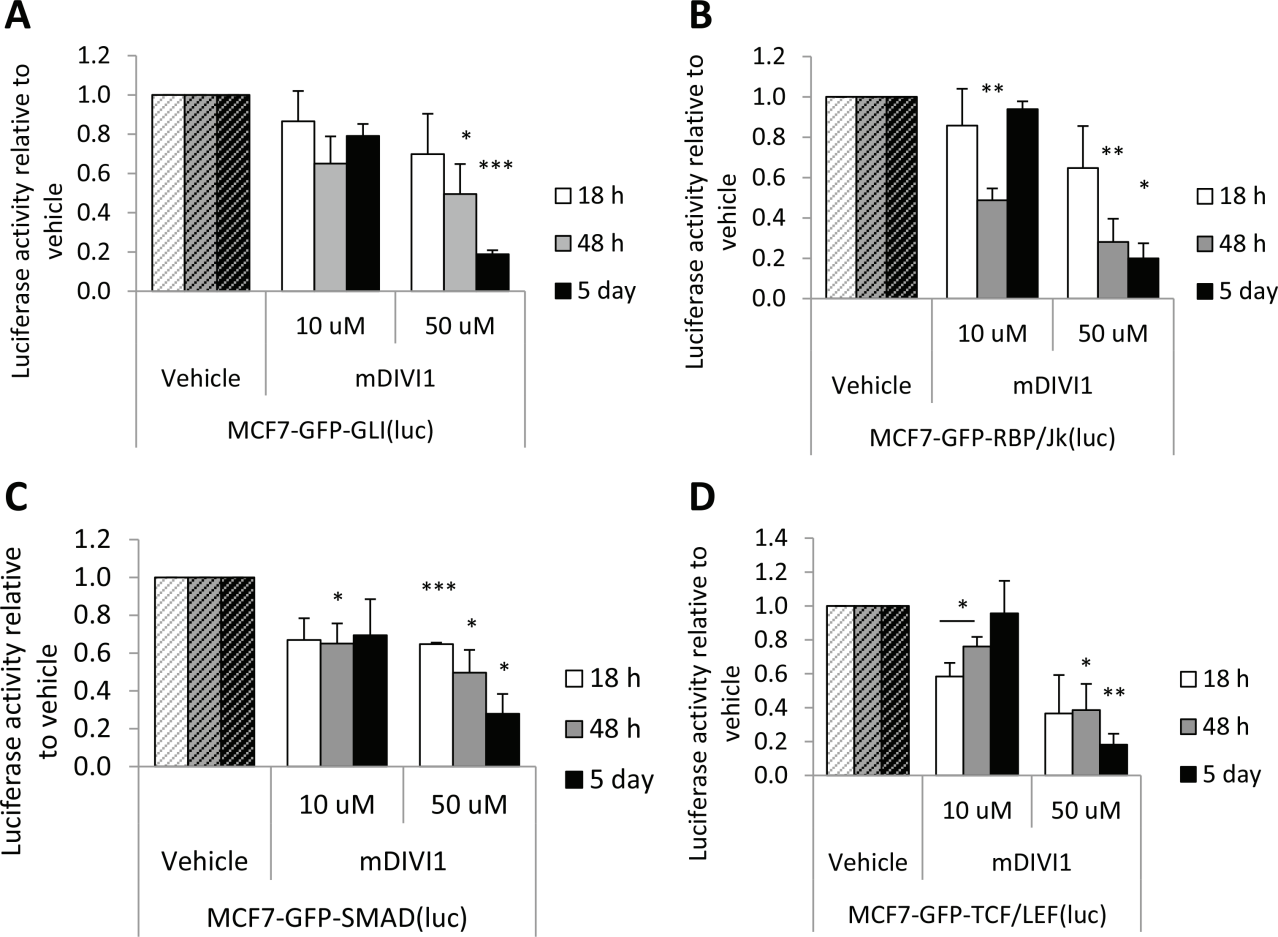
mDIVI1 treatment significantly inhibits stem-related signalling in MCF7 cells (**A**) Hh/GLI (**B**) Notch/Rbp/Jk (**C**) TGFβ/SMAD and (**D**) Wnt/TCF/LEF signaling were significantly inhibited after treatment with mDIVI1 at most time points assessed, particularly after exposure to 50 µM mDIVI1. Data represented as MEAN ± SEM. One sample *t* test, ^*^p value < 0.05, ^**^p value < 0.01 and ^***^p value < 0.001. *N* = 3

Mitochondrial fusion and fission may also have an impact in signalling pathways that regulate stem cell proliferation and survival [17]. Thus, we next decided to determine how several signalling pathways that regulate stemness were affected by mDIVI1 exposure. For that purpose, a range of reporter MCF7-GFP cell lines were generated, including MCF7-GFP-GLI(luc), MCF7-GFP-Rbp/Jk(luc), MCF7-GFP-SMAD(luc) and MCF7-GFP-TCF/LEF(luc), to asses Hedgehog signalling, Notch signalling, TGFβ signalling and Wnt signalling respectively. These cell lines were exposed to either mDIVI1 or vehicle and assessed for luciferase activity. For the first time, we show that mDIVI1 treatment significantly inhibited all stem-related signalling pathways in a dose-dependent manner at most of the time points evaluated. Particularly, 50 µM mDIVI1 strikingly caused the inhibition of the Hedgehog/GLI pathway, the Notch/Rbp pathway, the Wnt/TCF pathway and the TGFβ/SMAD pathway 2 and 5 days after the start of the treatment (Figure 7).

Moreover, STAT3 (-1.83), EphA2 (-1.74) and BMP7 (-47.17), all proteins involved in signalling pathways associated with cancer stem cell activities, were also found to be down-regulated in the proteomics datasets. Ephrin receptor signalling was specifically found to be significantly altered in mDIVI1-treated MCF7 cells compared to vehicle, with a negative z score indicating a slight inhibition of this pathway (Table 2). Likewise, TGFβ was identified by IPA as an upstream regulator predicted to be downregulated (*z* score = −1.934).

In summary, mDIVI1 inhibits all studied signalling pathways related with stemness in MCF7 cancer cells, confirming a suppression of the stem-like phenotype in these cells after treatment.

### MDIVI1 increases CD44^+^/CD24^−^ population of MCF7 cells at high concentrations

**Figure 8:**
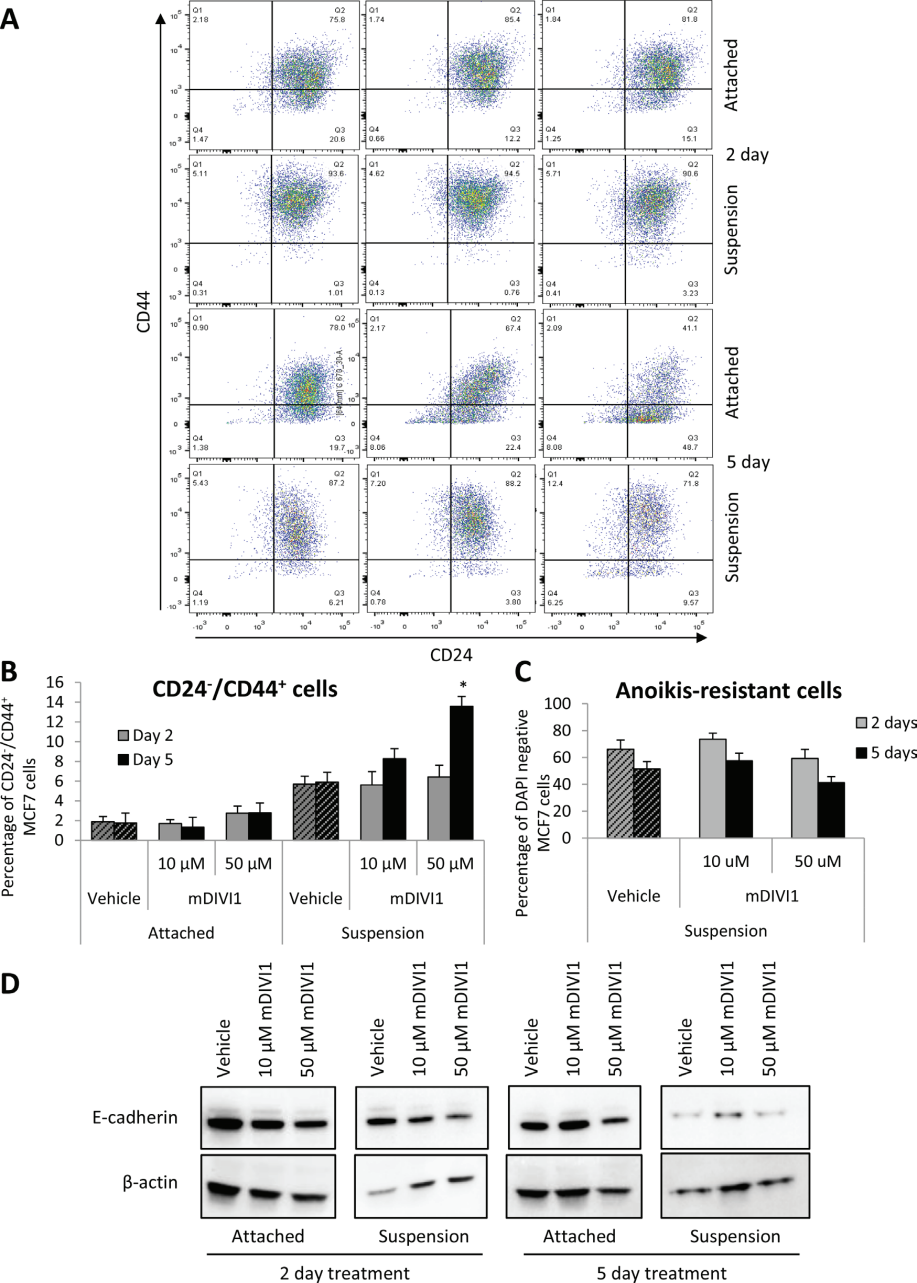
mDIVI1 treatment significantly increases the CD24^−^/CD44^+^ population of MCF7 cells at high concentrations (**A**) Representative graph showing CD24/CD44 staining of MCF7 cells treated with either vehicle or mDIVI1 and grown as either monolayers or in suspension for 2 or 5 days. (**B**) CD24^−^/CD44^+^ subpopulation of MCF7 cells significantly increased after treatment with 50 µM of mDIVI1 in suspension. (**C**) The percentage of anoikis-resistant cells did not significantly differ in mDIVI1 treatments from the vehicle treatment. One way ANOVA, Dunnett’s multiple comparisons test, ^*^p value < 0.05, ^**^p value < 0.01 and ^***^p value < 0.001. *N* = 4 (**D**) Immunoblot analysis of MCF7 cells treated with mDIVI1 revealed a decrease of E-cadherin expression after mDIVI1 treatments.

In human breast cancers, CSCs were first identified by the profile of expression of the cell surface marker CD24^−^/CD44^+^ [18]. Thus, we next determined the expression levels of these CSC markers in MCF7 grown either as monolayers or under anchorage-independent conditions and exposed to mDIVI1 or vehicle. Surprisingly, the abundance of the CD24^−^/CD44^+^ subpopulation of MCF7 cells was not reduced after exposure to 10 or 50 µM mDIVI1 compared to vehicle in cells grown as monolayers. Moreover, treatment of MCF7 cells in suspension with mDIVI1 unexpectedly increased the amount of CD24^−^/CD44^+^ cells in a dose-dependent fashion (Figure 8A and 8B). Thus, mDIVI1 exposure does not alter the proportion of CD24^−^/CD44^+^ MCF7 CSCs grown as monolayers. However, under anchorage-independent growth conditions, the quantity of CD24^−^/CD44^+^ MCF7 cells increases in the mDIVI1 treatments in a dose-dependent manner.

It has been recently described that breast CSCs can exist in distinct states, a mesenchymal-like state and an epithelial-like state, being the first one characterised for its expression of CD24^−^/CD44^+^, with a mesenchymal-like gene-expression profile and primarily quiescent [18]. Thus, we hypothesised that the increase in CD24^−^/CD44^+^ MCF7 cells in suspension after treatment with mDIVI1 could respond to a change in these cells from an epithelial towards a more mesenchymal phenotype. We therefore searched for the expression of epithelial markers in MCF7 cells treated with either vehicle or mDIVI1. Immunoblot analysis of MCF7 cells treated with mDIVI1 revealed a reduction in the expression of E-cadherin after mDIVI1 treatment (Figure 8D). Decrease in the expression of other epithelial markers was observed also in the proteomics datasets, including EPCAM (−1.58), ZO-1 (−1.62), keratins 8 and 18 (−5.24 and −12.21), CELSR2, involved in loss of polarization (−1.6), F11 receptor (−1.70), junction plakoglobin (−1.65) and desmoplakin (−4.58), although other epithelial markers such as collagen type IV was found to be upregulated by 1.9 fold. However, no mesencymal markers were identified as upregulated by proteomics. Notably, amongst the altered pathways identified by IPA in the mDIVI1-treated cells were pathways involved in cell to cell interaction and cell junctions such as remodeling of epithelial adherens junctions or epithelial adherens junctions signalling, tight junction signalling, gap junction signalling or FAK, integrin or paxillin signalling (Table 2). Thus, it seems mDIVI1 exposure induces the acquisition of a CD24^−^/CD44^+^ phenotype in MCF7 cells in suspension with loss of epithelial markers although no complete epithelial-to-mesenchymal transition is observed.

The DAPI staining that was performed in parallel to CD24^−^/CD44^+^ staining to be used as quantification of viable cells, also revealed that there were no significant differences in cell viability between vehicle-treated cells and mDIVI1-treated cells under anchorage-independent conditions (Figure 8C). That is, mDIVI1-treated cells are not propagating as efficiently as vehicle-treated cells do, as observed in the tumorsphere formation assay, however they are more viable after mDIVI1 treatment in suspension than under attachment conditions. One possible explanation to that observed behaviour is that they are becoming more quiescent. In fact, IPA analysis identifies cell cycle, M phase, cytokinesis, cell viability or cell proliferation as biological functions that are predicted to be decreased in mDIVI1-treated MCF7 cells compared to vehicle (Table 3). In addition, treatment with 50 µM mDIVI1 showed frequent aberrations associated with failure of cytokinesis, which could be seen by DAPI immunofluorescence staining as an increase in binucleated cells (data not shown). Such phenomenon is typical of anti-mitotic drugs.

## DISCUSSION

**Figure 9:**
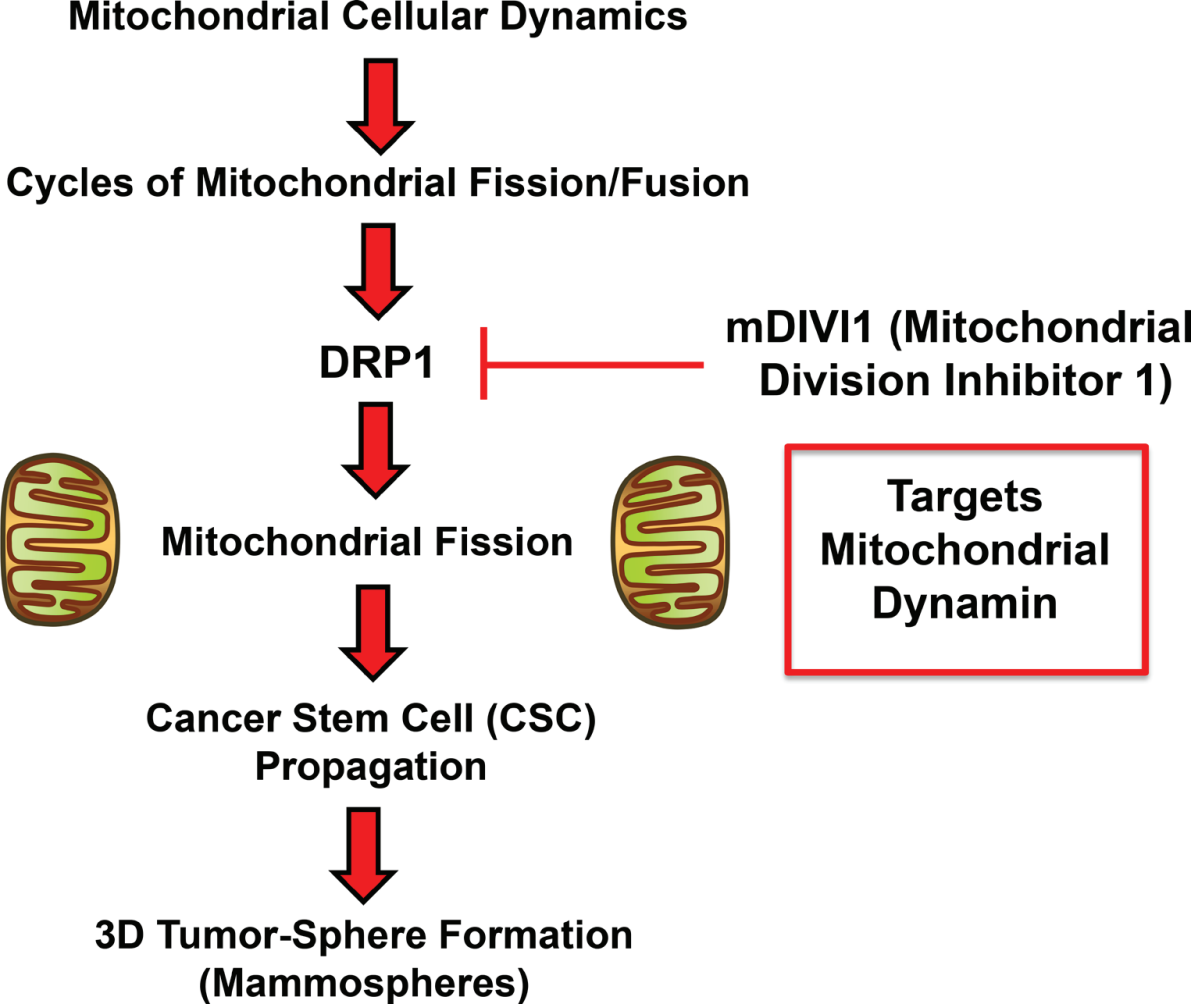
Schematic diagram summarizing our approach using mDIVI1 to investigate the role of mitochondrial fission/fusion in CSC propagation Note that mDIVI1 inhibits DRP1, a mitochondrial-specific dynamin protein. See text for further details.

Recent findings show that mitochondrial morphology and function contribute to a stem-like phenotype, implying that mitochondrial fission and fusion are central players in CSC behaviour. The balance in mitochondrial dynamics is controlled by a small cohort of regulators, such as DRP1. mDIVI1 is a small molecule inhibitor of DRP1 used in research for inhibiting mitochondrial fission (Figure 9). Emerging evidence demonstrates the implication of mitochondrial fission and in particular DRP1 in the tumorigenicity and maintenance of stem-like cells [7, 8]. However, a comprehensive study of the effects of mDIVI1 on CSC behaviour is still needed. Here, we tested the hypothesis that mDIVI1, through inhibition of mitochondrial fission, would induce loss of oxidative phosphorylation, therefore reducing the propagation of CSC, as they rely on their mitochondrial metabolism for their survival [11–13].

Our results show that mDIVI1-induced inhibition of DRP1 in MCF7 cells has a positive effect on their mitochondrial mass and production of mitochondrial reactive oxygen species, and that has a negative impact on mitochondrial metabolism. Mitochondrial fission plays a role in mitophagy, as it facilitates the elimination of defective organelles via isolation of selective parts of the mitochondria from the mitochondrial network. Therefore it is not surprising that after inhibiting DRP1, a slight increase in mitochondrial mass is observed. A few studies associate DRP1 and mitochondrial oxidative stress. Mitochondrial ROS levels are lower in DRP-overexpressing cells whereas those in a DRP1 mutant that inhibits mitochondrial fission are increased [14]. Moreover, when stress-induced hyperfusion of mitochondria happens in differentiated tissue, it increases mitochondrial ROS levels [19]. mDIVI1 also causes mitochondrial dysfunction and subsequent cell apoptosis in a DRP1-dependent manner in chemoresistant breast cancer cells [20]. In the current study, we show that mDIVI1-induced DRP1 inhibition alters the exctracellular acidification rate and oxygen consumption rate of MCF7 cells, transforming them in less aerobic, less metabolically active cells. mDIVI1 treatment seems to also have a big impact on lipid metabolism.

Previous studies performed in our lab strongly indicate that mitochondrial function has an implication in the propagation of CSCs [11–13]. We show here that mDIVI1-induced reduction in the respiration of MCF7 cells also impedes their propagation under anchorage-independent conditions. In addition, we show for the first time that mDIVI1 treatment decreases tumorsphere forming capacity in melanoma and lung cancer cell lines. However, the sphere assay has several weaknesses as even when plating them at a limiting dilution, individual spheres can be formed out of aggregation of cells rather than be generated through clonal expansion. Furthermore, more differentiated cells can also exhibit sphere-forming capability, and because tumorspheres themselves are heterogeneous, experiments using them should be interpreted as studies of mixed cell populations enriched for stem-like cells, not totally purified CSCs [21]. Taken together, these considerations made us use other approaches to asses CSC phenotype and activity, including the evaluation of several stemness-related pathways or the migratory ability of mDIVI1-treated MCF7 cells. Indeed, via generation of several reporter cell lines, we confirmed that mDIVI1 treatment was able to inhibit stemness-related signalling in MCF7, and mDIVI1 had also a negative effect on MCF7 cell motility.

Increased cell motility and invasion seem to require mitochondrial elongation and trafficking to the periphery of the cell. Several reports link mitochondrial localisation and cell motility by describing a redistribution of mitochondria toward the leading edge of cells during persistent migration. DRP1 silencing inhibits lamellipodia formation, a key step for cancer metastasis, by suppressing recruitment of mitochondria to those regions, and therefore decreasing the metastatic potential of breast cancer [22]. In order to be transported around the cells, mitochondria need to be transformed into small units, free from a tight network organization. Is during that process that DRP1 may play a role. Mitochondria might be needed at the leading edge as a source of energy, for calcium signalling, for the stabilisation of microtubules by ATP or even for the production of fatty acids and eicosanoids for membrane dynamics in the proximity of focal adhesions [23]. Our results show that treatment with mDIVI1 actually alters these processes, including oxidative phosphorylation and ATP production, lipid metabolism, and calcium and focal adhesion signalling (Table 2). Oxidative phosphorylation is actually required for the transfer of mitochondria to the cortical cytoskeleton. In respiration-deficient tumour cells, there is a lack of mitochondria in focal adhesion complexes, and invasion is impared [23]. Thus, via inhibition of mitochondrial fission and oxidative phosphorylation, mDIVI1 avoids distribution of mitochondria to the leading edge of the cell, and migration is impeded.

Indeed several studies point out that actin polymerization promotes mitochondrial fission. In addition, actin-depolymerizing drugs inhibit the recruitment of DRP1 to mitochondria and mitochondrial length is reduced [24]. Our proteomics results also show that one of the top canonical pathways affected by mDIVI1 is the actin cytoskeleton signalling.

Unexpectedly, we also found an increase in the abundance of CD24^−^/CD44^+^ cells, which is a marker of CSC activity. Breast CSCs can exist in reversible heterogeneous states, a more epithelial-like state, and a more mesenchymal-like state, characterised for its expression of CD24^−^/CD44^+^, with marked reduction of oxygen consumption and increased quiescence [18]. However, although there was a clear reduction of epithelial markers after exposure to mDIVI1, as well as a reduction of mitochondrial respiration, we could not identify an over-expression of mesenchymal markers in mDIVI1-treated MCF7 cells. Interestingly, during pluripotency reprogramming, epithelial-like cells display more fragmented mitochondria, indicating that mitochondrial fission is critical for the acquisition of pluripotency [9].

Finally, compared to vehicle-treated cells, mDIVI1-treated cells seem to survive more in suspension than under attachment conditions, which could be related to the acquisition of a quiescent state. An exhaustive analysis of the cell cycle would be strongly advised to be able to tell whether these cells are actually acquiring a quiescent phenotype or are undergoing senescence. Our proteomics datasets did not identify many markers of senescence. Only LAMP1 was found to be upregulated in mDIVI1-treated cells compared to vehicle (2.83), although it is also involved in the processes of autophagy and mitophagy.

In summary, mDIVI1 has a negative impact on the anchorage-independent growth of MCF7 cells, on their migratory capacity and their signalling pathways related to stemness. However, it seems that mDIVI1 selects for a phenotype with loss of epithelial markers and features, such as cell to cell contact establishment, and in suspension enriches the CD24^−^/CD44^+^ population of cells. Several options could explain that behaviour. The first one could be that CD24^−^/CD44^+^ expression is not representative of CSC activity in this scenario of mitochondrial fission inhibition, as for instance DRP1 effects on CSC behaviour are downstream of CD44, or mDIVI1 has a direct effect in CD24 loss or CD44 acquisition that does not have an impact on CSC survival. Another explanation would be that MCF7 cells enrich their CD24^−^/CD44^+^ expression profile to counteract mDIVI1 effects, without being able to reverse the inhibition of the CSCs phenotype (tumorsphere formation efficiency, migration and stemness signalling) and therefore depleting CSCs.

Metabolism is materialising as a promising area for cancer treatment [25]. Nevertheless, the notion of targeting cancer mitochondrial metabolism or organelle-driven adaptation is still underdeveloped. This study has been done in an effort to contribute to that concept, as a better understanding of processes engaged in the regulation of mitochondrial dynamics and their significance for CSC maintenance and propagation will provide us with the instruments to eventually alter them, offering new possible therapeutic approaches. It appears that CSCs have adopted metabolic control mechanisms to increase their survival and proliferation. In that scenario, mitochondrially-targeted drugs may represent promising new agents to interfere with tumour adaptation, ultimately eliminating CSCs [26]. The fact that mDIVI1 acts as a cytoprotective agent in non-tumour cells and that it would allow a reversible manipulation of mitochondrial morphology highlights its importance as a putative anti-cancer agent.

## MATERIALS AND METHODS

### Cell culture

Human MCF7 breast cancer cells were purchased from ATCC and maintained in complete media: DMEM (D6546, Sigma) supplemented with 10% fetal bovine serum (F7524, Sigma), 100 units/ml of penicillin, 100 µg/ml, streptomycin (P0781, Sigma) and 1% Glutamax (#35050087, Life Technologies). For all experiments, cells were incubated in a 5% CO_2_ atmosphere at 37° C.

### Chemicals

mDIVI1 or 3-(2,4-Dichloro-5-methoxyphenyl) 2,3-dihydro 2-thioxo-4(1H) quinazolinone (sc-215291, Santa Cruz), an inhibitor of mitochondrial division DRP1 and dynamin I, was used in this study at the concentrations indicated. Vehicle-treated cells (DMSO 0.5%) were analysed in all experiments.

### Prestoblue viability assay

Cell viability was measured using the resazurin-based PrestoBlue reagent (A-13261, ThermoFisher Scientific). Briefly, 5 × 10^3^ MCF7 cells were seeded into 96-well black plates. When cells were attached mDIVI1 or vehicle were added to the cells. After 2 or 5 days, PrestoBlue reagent was added to the cells and incubated for 2 hours. Plates were finally read using a FluoStar Omega plate reader (BMG Labtech) at an excitation wavelength of 544 and an emission wavelength of 590 nm. Background measurements were subtracted from all values.

### Mitochondrial mass quantification

To measure mitochondrial mass, cells were stained with MitoTracker Deep Red (M22426, Invitrogen). Briefly, 2 × 10^5^ MCF7 cells per well were seeded in 6-well plates. When cells were attached, mDIVI1 and vehicle treatments were added for 2 days or 5 days in triplicate. Cells were then incubated for 15 min at 37° C with the 10 nM Mitotracker Deep Red probe diluted in PBS (D8662, Sigma). All subsequent steps were performed in the dark. Cells were washed in PBS, harvested, and re-suspended in PBS. Mitotracker signal was quantified as mean fluorescent intensity of the viable cell population in a Fortessa flow cytometer (BD Bioscience). Results were analyzed using FlowJo software.

### Mitochondrial staining

One hundred thousand MCF7 cells were seeded onto coverslips in 12-well plates. When cells were attached, mDIVI1 and vehicle treatments were performed in triplicate. After 2 days and 5 days, mitochondria were labeled by incubating cells for 15 min at 37° C with 25 nM of MitoTracker DeepRed (M22426, Invitrogen) diluted in PBS (D8662, Sigma). Then, cells were washed with PBS, fixed in 2% paraformaldehyde (28908, Thermo Scientific) in PBS for 30 minutes at room temperature and mounted with ProLong^®^ Gold Antifade Mountant reagent with DAPI (P36935, Invitrogen, Inc.). Immunofluorescence pictures were taken in a Leica gated Stimulated Emission Depletion Microscopy (gSTED) with additional confocal and multi-photon illumination (room rg106).

### Levels of reactive oxygen species and mitochondrial superoxide

For both assays, 2 × 10^5^ MCF7 cells were seeded in 35 mm plates. When cells were attached, either vehicle or mDIVI1 were added to the cells for 24, 48 hours or 5 days in triplicate. Reactive oxygen species (ROS) production was measured using CM-H_2_DCFDA (C6827, Invitrogen). Briefly, cells were incubated for 20 minutes at 37° C with 1 µM CM-H_2_DCFDA diluted in PBS and then placed in complete media for 20 minutes at 37° C in the dark, to render the dye fluorescent, according to the manufacturer. Mitochondrial superoxide was measured using MitoSOX Red Mitochondrial Superoxide Indicator (M36008, ThermoFisher Scientific). Briefly, cells were incubated for 10 minutes at 37° C in the dark with 5 µM MitoSOX diluted in PBS, according to the manufacturer. ROS signal and MitoSOX signal was quantified as mean fluorescent intensity of the viable cell population in a Fortessa flow cytometer (BD Bioscience). Results were analysed using FlowJo software.

### Extracellular flux analysis and bradford assay

Extracellular acidification rate (ECAR) and oxygen consumption rate (OCR) were measured in a XF96 Extracellular Flux Analyzer (Seahorse Biosciences). Briefly, 1 × 10^4^ MCF7 cells per well were seeded in XF96 plates and incubated with complete medium. When cells were attached, mDIVI1 and vehicle treatments were added. Six replicates were run for each condition. After 48 hours, un-buffered DMEM XF medium supplemented with 2 mM glutamine (pH 7.4) was added to the cells, and placed in a 37° C CO_2_-free incubator for 1 hour. Ten mM glucose, 1 μM oligomycin and 100 mM 2-deoxyglucose (2-DG) were injected into the media at different time points and ECAR was measured. Likewise, ECAR and OCR were quantified using un-buffered DMEM XF medium supplemented with 2 mM glutamine, 2 mM sodium pyruvate and 10 mM glucose. One μM oligomycin, 0.9 μM FCCP and 1 μM rotenone and antimycin A were injected into the media at different time points and OCR and ECAR were measured.

Cells were finally lysed with 0.1 M NaOH and protein lysate was subsequently stained with Quick Start™ Bradford 1× Dye Reagent (500–0205, BioRad) to normalise results. All parameters were calculated according to manufacturer.

### Tumorsphere formation assay

A single cell suspension was prepared using enzymatic (1× Trypsin-EDTA, T3924, Sigma Aldrich), and manual disaggregation (25 gauge needle) [27]. Cells were plated at a density of 5000 cells/well in tumorsphere media (DMEM-F12/B27/EGF (20ng/ml)/Pen-Strep) in non-adherent conditions, in 6-well plates coated with (2-hydroxyethylmethacrylate) (poly-HEMA, P3932, Sigma) in the presence of mDIVI1 or vehicle. After 5 days of culture in a humidified incubator at 37° C, tumorspheres bigger than 50 μm were counted using an eye piece graticule.

### Scratch assay

A hundred thousand MCF7 cells per well were seeded in 12-well plates. When cells were attached, a horizontal scratch was performed using the tip of a pipette. Immediately after, the media was removed and either fresh vehicle-containing media or mDIVI1-containing media were added to the cells for 48 hours. Four replicates were used for each condition. Cells were incubated in a 5% CO_2_ atmosphere at 37° C in an Incucyte ZOOM System (Essen Bioscience). The wound closure was monitored by taking pictures of each well every 4 hours. At the end of the treatment, pictures of the wells at 0 and 48 hours were analysed using ImageJ software to calculate wound closure according to the following formula:

$$\text{Percentage wound closure}=\left[\left({\text{wound area}}_{0\text{h}}-{\text{wound area}}_{48\text{h}}\right)/{\text{wound area}}_{\text{0h}}\right]\times 100$$

### Luciferase assay

The Cignal Lenti reporter assay (luc) was used to monitor the activity of several signalling pathways in MCF7-GFP cells as explained previously [28]. Luciferase Assay System (E1501, Promega) was performed according to manufacturer’s instructions. 1 × 10^4^ MCF7 were seeded in black-walled 96 well plates. When cells were attached, drug treatments were added for 18, 48 hours and 5 days. After treatment, Luciferase Assay was performed according to manufacturer’s instructions and light signal was acquired in the Xenogen VivoVision IVIS Lumina. Results were normalized by SRB staining of cells grown in 96-well plates in parallel.

### Sulforhodamine b (SRB) assay

SRB (S9012, Sigma) measures total biomass by staining cellular proteins. After treatment, cells were fixed in 10% tricloroacetic acid (T9159, Sigma) for 1 hour at 4° C, stained with SRB (S9012, Sigma) for 15 minutes, and washed 3 times with 1% acetic acid (27225, Sigma). The incorporated die was solubilized with 10 mM Tris Base, pH 8.8 (T1503, Sigma). Absorbance was spectrophotometrically measured at 562 nm in a FluoStar Omega plate reader (BMG Labtech). Background measurements were subtracted from all values.

### CD24/CD44 expression

Four hundred thousand MCF7 cells were seeded either in either a regular 10 cm dish or in five 15 cm dishes coated with (2-hydroxyethylmethacrylate) (poly-HEMA, P3932, Sigma) in the presence of mDIVI1 or vehicle for 2 days or 5 days. Following mDIVI1 treatment, MCF7 cells grown either in suspension or as monolayers were analysed for their expression of CD24 and CD44. The surviving fraction after 2 days and 5 days of growth was analyzed by FACS. MCF7 cells were either trypsinised or spun down and trypsinised and incubated with CD24 (IOTest CD24-PE, Beckman Coulter) and CD44 (APC mouse anti-human CD44, 559942, BD Pharmingen) for 15 minutes on ice. Cells were then rinsed twice in PBS, spun down, and resuspended with DAPI dye (D1306, Molecular probes) at 3 µM in PBS for 10 minutes. Samples were then analyzed by FACS (Fortessa, BD Bioscence). Only the live cell population, identified using the DAPI staining, was analyzed for CD24/CD44 expression. Data were analyzed using FlowJo software. Only those cells expressing CD44 that did not express CD24 were considered to be the CD24^−^/CD44^+^ CSC subpopulation.

### Western blotting

Two million MCF7 cells were seeded either in regular 15 cm dishes or in five 15 cm dishes coated with (2-hydroxyethylmethacrylate) (poly-HEMA, P3932, Sigma) in the presence of mDIVI1 or vehicle for 2 days or 5 days. At the end of the treatment, cells in suspension were spun down and lysed and attached cells were directly lysed in RIPA lysis buffer (R0278, Sigma) containing proteinase inhibitors (05 892 970 001, Roche) and kept at 4° C for 20 minutes with rotation. Lysates were cleared by centrifugation for 10 minutes at 10,000 xg and supernatants were collected. Equal amounts of protein lysate, as determined by using the BCA protein assay kit (23225, Pierce) were diluted in SDS sample buffer and dry-boiled for 5 minutes before being separated by SDS-PAGE using 4–20% gels (456–1094, Biorad). Samples were then blotted onto nitrocellulose membranes (170–4159, Biorad), blocked in 5% milk in TBS-Tween 20 (P9416, Sigma) for 1 hour and probed with antibodies against E-cadherin (ab8995, Abcam) or β-actin (a2228, Sigma). Bound antibodies were detected using a horseradish peroxidase-conjugated secondary antibody (ab6789 and ab6721, Abcam) and signal was obtained using Supersignal West Pico chemiluminiscent substrate (34087, ThermoScientific). Pictures were taken in a ChemiDoc XRS with Image Lab Software (BioRad).

### Label-free semi-quantitative proteomics

#### Chemicals and sample preparation.

Formic acid, trifluoroacetic acid, ammonium formate (10 M), ammonium bicarbonate TCEP (Tris (2-carboxyethyl)phosphine hydrochloride), MMTS (Methyl methanethiosulfonate) and trypsin were all obtained from Sigma. HPLC gradient grade acetonitrile was obtained from Fisher Scientific. Briefly, 2 × 10^6^ MCF7 cells were seeded in 15 cm plates until cells were attached. Cells were then treated with 10 **µ**M mDIVI1. As control, vehicle-treated cells were processed in parallel. After 48 hours of treatment, cells were lysed in RIPA buffer (R0278, Sigma) and kept at 4° C for 20 minutes with rotation. Lysates were cleared by centrifugation for 10 minutes at 10,000 × g and supernatants were collected and kept frozen at −80° C.

#### Protein digestion

Lysate samples were thawed to room temperature and their concentrations equalised to 1 µg/µL (50 µL volume) with RIPA buffer, and further processed for trypsin digestion by sequential reduction of disulphide bonds with TCEP and alkylation with MMTS. Briefly, 1 µL benzonase (Novagen) was added to the 50 µL aliquot and placed on ice for 15 minutes. The sample was then taken to dryness using a SpeedVac, and resuspended in 22.5 µL trypsin reaction buffer (40 mM ammonium bicarbonate and 9% acetonitrile). One µL of 50 mM TCEP solution was added to each sample, mixed briefly and placed on a heater block at 60° C for 60 minutes. After cooling to room temperature, 0.5 µL of 200 mM MMTS solution was added to each sample and allowed to react for 15 minutes. Trypsin was added in two waves to ensure efficient digestion of the sample. Firstly, 20 µg of sequencing grade trypsin was resuspended in 1800 µL of trypsin reaction buffer; 225 µL of this solution were added to each sample for digestion, and the reactions were left at 37° C overnight with shaking (600 rpm). The following morning, a further aliquot of trypsin was added. Two ml of trypsin reaction buffer was added to 20 µL of sequencing grade trypsin; 250 µL of this solution were added to each of the digest samples from overnight, and the reactions were left at 37° C for 4 hours with shaking (600 rpm). Thirty-five µL 10% formic acid were added to the 500 µL digest sample (0.7% final concentration of formic acid) to stop the digestion. The digested solution was diluted in 7.5 mL of acetonitrile containing 0.3% formic acid.

#### HILIC solid phase extraction (SPE) of peptides

PolyhydroxyethylA SPE 12 µm, 300A, 300mg cartridges (obtained from PolyLC) were used for the HILIC procedure. Prior to use, cartridges required an overnight soak in 50 mM formic acid followed by rinsing with water the following day. Cartridges were preconditioned with 2 mL of Buffer A (90% acetonitrile, 5 mM ammonium formate, pH 2.7) followed by 2 mL of Buffer B (5mM ammonium formate, pH 2.7) and finally re-equilibrated with 10 mL Buffer A. The diluted samples were loaded onto the cartridges and washed with a further 10 mL Buffer A. Finally, peptides were eluted in 1 mL Buffer C (9 parts Buffer B plus 1 part Buffer A) and the samples dried on a Speedvac to remove organic solvent prior to LC-MS/MS analysis.

#### LC-MS/MS analysis

Lyophilised digests were resuspended in 50 µL of 0.1% TFA to give an approximate concentration of 1 µg/µL. One µL injection volumes were used throughout resulting in an on-column peptide loading of approximately 1 µg per injection. Analysis was performed in quintuplicate for each sample. All LC-MS/MS analyses were performed on an LTQ Orbitrap XL mass spectrometer coupled to an Ultimate 3000 RSLCnano system (Thermo Scientific). One µL injection volumes were used throughout and samples loaded directly onto the analytical column, PepMap RSLC C18, 2 µm × 75 µm id × 50 cm (Thermo Scientific). The composition (v/v) of LC buffers were as follows; Buffer A - 99.9% water plus 0.1% formic acid and Buffer B −80% acetonitrile, 19.9% water and 0.1% formic acid. Peptides were loaded directly onto the column at a flow rate of 400 nl/min with an initial mobile phase composition of 1% B. The organic strength was increased linearly from 1% to 22.5% B over 22.5 minutes again at 400 nl/min, followed by an increase to 24.8% B over the next 2.6 minutes with a concomitant reduction in flow rate to 300 nl/min, and to 39% B over a further 14 minutes. A further increase to 60% B over the next 5 minutes was followed by a ramp to 95% B over 2.5 minutes where it was held for a further 2 minutes. The column was then allowed to re-equilibrate to 1% B for a total analysis time of 74 minutes. The mass spectrometer was instructed to perform data dependent acquisition on the top six precursor ions, which were measured in the Orbitrap FTMS detector over the mass range 370–1200 m/z, at a nominal resolution of 60,000. MS/MS spectra were acquired in the ion trap under CID conditions with normalized collision energy of 35, isolation width of 3 Th, Q value of 0.25 and 30 ms activation time. Gas-phase fractionation was performed on the five replicate injections such that MS/MS data was collected for precursor ion range 370–494 m/z Injection 1, 494–595 m/z Injection 2, 595–685 m/z Injection 3, 685–817 m/z Injection 4 and 817–1200 m/z Injection 5.

#### Analysis

Xcalibur raw data files acquired on the LTQ-Orbitrap XL were directly imported into Progenesis LCMS software (Waters Corp) for peak detection and alignment. Data were analysed using the Mascot search engine. Five replicates were analysed for each sample type (*N* = 5).

### Ingenuity pathway analyses

Pathway and function analyses were generated using Ingenuity Pathway Analysis (IPA) (Ingenuity systems, http://www.ingenuity.com), which assists with proteomics data interpretation via grouping differentially expressed genes or proteins into known functions and pathways. Pathways with a *z* score > 1.9 were considered as significantly activated, and pathways with a *z* score < −1.9 were considered as significantly inhibited.

### Statistical analyses

ANOVA was used for statistical comparison of three or more groups. For normalised data, one-sample *t* test was performed. All data are reported as mean ± standard deviation of the mean (SEM). All experiments were performed at least three times with reproducible results unless otherwise stated. *P* values lower than 0.05 were considered significant (^*^P < 0.05, ^**^P < 0.01, ^***^P < 0.001). Microsoft Excel was used to produce all graphs except FACS analysis graphs, in which case FlowJo was used.

## SUPPLEMENTARY MATERIALS FIGURE


