# Supplementary material for: Mitochondrial fission as a driver of stemness in tumor cells: mDIVI1 inhibits mitochondrial function, cell migration and cancer stem cell (CSC) signalling
Source: Oncotarget. 2018 Jan 19;9(17):13254–75. doi: 10.18632/oncotarget.24285 (PMC5862576; doi:10.18632/oncotarget.24285)
Supplement: Supplementary file 1 [file oncotarget-09-13254-s001.pdf]

Mitochondrial fission as a driver of stemness in tumor cells: mDIVI1 inhibits mitochondrial function, cell migration and cancer stem cell (CSC) signalling

SUPPLEMENTARY MATERIALS

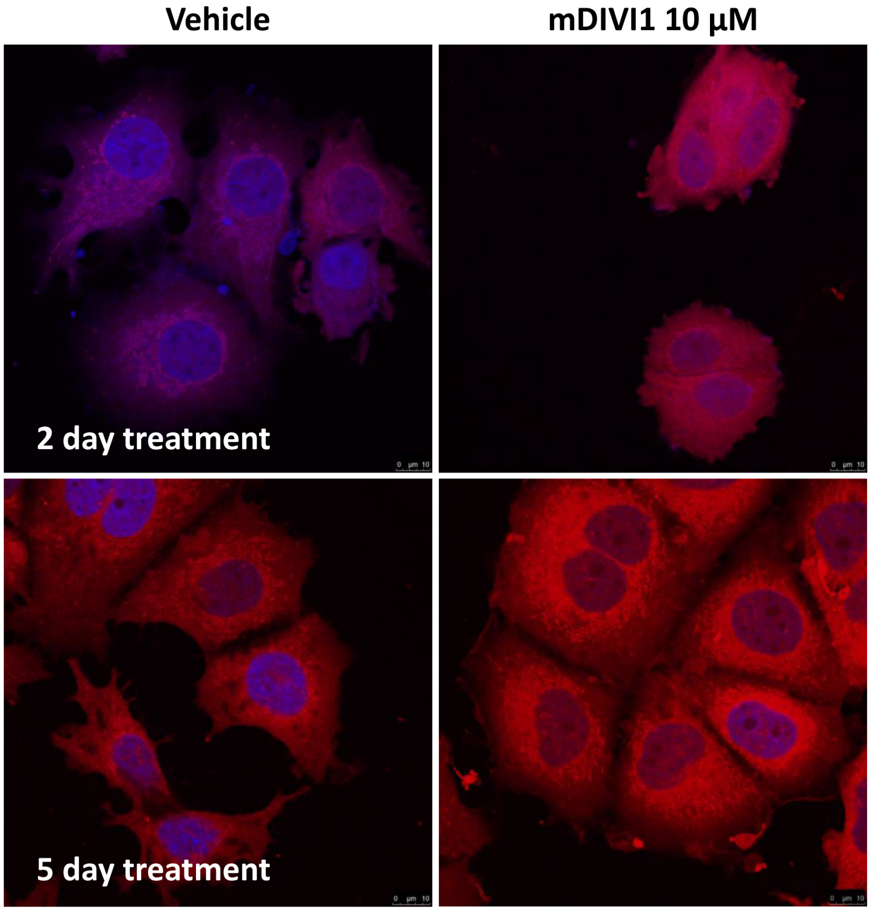

Supplementary Figure 1: Mitochondrial staining of MCF7 treated with either vehicle or 10 µM of mDIVI1 for 2 days or 5 days. mDIVI1 treatment appears to extend/disperse the perinuclear staining of MCF7 mitochondria compared to vehicle treatment and slightly increase mitochondrial caliber. Mitotracker Deep Red was used to stain mitochondrial mass and DAPI was used for counterstaining cell nuclei. Representative image of triplicate samples.
